# Editorial: Nutrient requirements and diabetes during pregnancy

**DOI:** 10.3389/fnut.2023.1270576

**Published:** 2023-10-25

**Authors:** Zheng Feei Ma

**Affiliations:** College of Health, Science and Society, University of the West of England, Bristol, United Kingdom

**Keywords:** iodine deficiency, maternal, pregnancy, diabetes, gestational

## Introduction

“Good health and wellbeing” is one of the Sustainable Development Goals (SDG) adopted by the United Nations in 2015 that highlights the importance of healthy lives and wellbeing for everyone at all ages. This include reducing the maternal mortality rates and preventable deaths under 5 years of age. One of the measures that can be taken to address these issues is to ensure that women meet the required nutrient recommendations, which may help to ensure the development of normal growth and good health of the infants. Hence, the nutritional status of women during pregnancy is critical for both the women and their infants. In particular, the first 1,000 days of life has been reported to be important for the prevention of non-communicable diseases in the later stage of life. Certain maternal conditions during pregnancy [e.g., iodine deficiency and gestational diabetes mellitus (GDM)] may lead to increased risks of some adverse health outcomes in infants (1). Therefore, the collection of the published papers in this Research Topic aimed to focus on the nutrient requirements and diabetes during pregnancy (Zhang et al.; Liu et al.; Gao et al.; Yan et al.)

One of the published papers included in this Research Topic focused on the maternal metabolism at different stages of pregnancy (Zhang et al.). The authors investigated the physiological hormones and nutrient metabolism characteristics of maternal serum and amniotic fluid. In their study, the dynamic temporal regulation of metabolic changes during pregnancy was associated with important maternal physiology during pregnancy (Zhang et al.). This because these metabolic changes are aim to protect the development of the fetus and ensure sufficient nutrient supply to the fetus. However, at the same time, it is important to note that some of these metabolic alterations including diabetes may have negative consequences on the infants. Therefore, there has been an increasing interest in exploring the effects of such metabolic alteration on the infant long-term health outcomes (2).

Another three published studies in this Research Topic were associated with GDM, which is one of the most common metabolic complications during pregnancy (Liu et al.; Gao et al.; Yan et al.). This because insulin resistance is mediated by increased level of fat deposits and placental hormones, leading to the blocking of insulin actions from binding to its receptors (3). The prevalence of GDM has been reported to range from 1 to 14%. In addition, other factors including obesity and physical inactivity are associated with higher insulin resistance, which leads to increased prevalence of GDM (4). Infants born to mothers who are diagnosed with GDM may have a higher risk of becoming obese or developing diabetes later in life (4).

Some micronutrient deficiencies during pregnancy have been associated with adverse pregnancy and infant outcomes. One of the common micronutrient deficiencies reported in pregnant women is iodine deficiency (5, 6). Iodine deficiency has been reported to affect about 30% of the world populations (7). For example, children of women with mild iodine deficiency during pregnancy were reported to have lower scores in reading accuracy, comprehension and verbal IQ than those of women with sufficient iodine status during pregnancy (8, 9). If left untreated, mild iodine deficiency during pregnancy may progress to severe iodine deficiency, which can lead to even greater harm and adverse obstetric outcome including increased risk of pregnancy loss and infant mortality (10).

Another common micronutrient deficiency during pregnancy is iron deficiency. There are up to 38% of women during pregnancy who reported to have anemia, with increased prevalence in low-income countries. In women with GDM, there is an association with intrauterine fetal hypoxia, which limits the oxygen supply to the fetus and may potentially increase the risk of iron deficiency in the fetus. This occurs as fetal erythropoiesis (production of red blood cells) increases, sometimes exceeding the system's capacity to adequately supply iron (11). Therefore, it is imperative to ensure that women are getting enough nutrients during pre-pregnancy and throughout pregnancy to prevent such adverse effects.

Therefore, it is important to note that these specific maternal conditions such as nutrient deficiencies and GDM are associated with higher risk of maternal and infant complications. In addition, the fetus is dependent on the maternal nutrition for normal growth and development. Therefore, good maternal nutrition plays an important in ensuring healthy pregnancy and infant outcomes.

## Call to action

Balancing nutrient requirements and managing diabetes during pregnancy is a multifaceted task. In addition, one of the public health initiatives should advocate for accessible and comprehensive support for pregnant women, especially with diabetes, to ensure that they receive the necessary support and resources to foster a healthy future for themselves and their newborns. Other public health initiatives include strategies to increase awareness of maternal nutrition status in women. The examples of nutrition information that may be interested to pregnant women are healthy eating, breastfeeding, heart burn, morning sickness and weight management. This is because increased awareness of maternal nutrition status may help them make better decisions and changes in their daily lives to protect their own health and fetal health (12).

## Author contributions

ZM: Writing—original draft, Writing—review and editing.

## References

[1] Ma ZF Zhou H Ma J Lu Y Pan B. Prevalence of diabetes mellitus and hypertension during pregnancy in eastern China after the implementation of universal two-child policy. Int J Diabetes Dev Ctries. (2021) 41:221–7. 10.1007/s13410-020-00872-x

[2] Parrettini S Caroli A Torlone E. Nutrition and metabolic adaptations in physiological and complicated pregnancy: focus on obesity and gestational diabetes. Front Endocrinol. (2020) 11:611929. 10.3389/fendo.2020.611929 33424775 PMC7793966

[3] Carr DB Gabbe S. Gestational diabetes: detection, management, and implications. Clin Diabetes. (1998) 16:4–12.

[4] Etminan-Bakhsh M Tadi S Hatami M Darabi R. Prevalence of gestational diabetes mellitus and its associated risk factors in Boo-Ali Hospital, Tehran. Galen Med J. (2020) 9:e1642. 10.31661/gmj.v9i0.1642 34466559 PMC8344143

[5] Yu Z Zheng C Zheng W Wan Z Bu Y Zhang G. Mild-to-moderate iodine deficiency in a sample of pregnant women and salt iodine concentration from Zhejiang province, China. Environ Geochem Health. (2020) 42:3811–8. 10.1007/s10653-020-00640-0 32596780

[6] Zhou H Lu Y Pan B Zhao Q Ma ZF. Iodine deficiency as assessed by neonatal TSH in a sample of mother-and-newborn pairs in Jiangsu Province, China. Biol Trace Elem Res. (2021) 199:70–5. 10.1007/s12011-020-02135-6 32253700

[7] Hatch-Mcchesney A Lieberman HR. Iodine and iodine deficiency: a comprehensive review of a re-emerging issue. Nutrients. (2022) 14:3474. 10.3390/nu14173474 36079737 PMC9459956

[8] Skeaff SA. Iodine deficiency in pregnancy: the effect on neurodevelopment in the child. Nutrients. (2011) 3:265–73. 10.3390/nu3020265 22254096 PMC3257674

[9] Bath SC Steer CD Golding J Emmett P Rayman MP. Effect of inadequate iodine status in UK pregnant women on cognitive outcomes in their children: results from the Avon Longitudinal Study of Parents and Children (ALSPAC). Lancet. (2013) 382:331–7. 10.1016/S0140-6736(13)60436-5 23706508

[10] Brough L. Global iodine status has improved: but we must not be complacent. Br J Nutr. (2017) 117:439–40. 10.1017/S0007114517000113 28215209

[11] Kiely ME Mccarthy EK Hennessy Á. Iron, iodine and vitamin D deficiencies during pregnancy: epidemiology, risk factors and developmental impacts. Proc Nutr Soc. (2021) 80:290–302. 10.1017/S0029665121001944 33988109

[12] Arrish J Yeatman H Williamson M. Midwives and nutrition education during pregnancy: a literature review. Women Birth. (2014) 27:2–8. 10.1016/j.wombi.2013.02.003 23562582

